# Efficacy of the Ubiquitous Spaced Retrieval-based Memory Advancement and Rehabilitation Training (USMART) program among patients with mild cognitive impairment: a randomized controlled crossover trial

**DOI:** 10.1186/s13195-017-0264-8

**Published:** 2017-06-06

**Authors:** Ji Won Han, Kyung Lak Son, Hye Jin Byun, Ji Won Ko, Kayoung Kim, Jong Woo Hong, Tae Hyun Kim, Ki Woong Kim

**Affiliations:** 1 Department of Neuropsychiatry, Seoul National University Bundang Hospital, 166 Gumiro, Bundanggu, Seongnamsi, Gyeonggido 463-707 Korea; 2 Department of Neuropsychiatry, Seoul National University Hospital, Seoul, Korea; 3 Department of Psychiatry, National Center for Mental Health, Seoul, Korea; 4 Department of Psychiatry, Seoul National University College of Medicine and Department of Brain and Cognitive Science Seoul National University College of Natural Sciences, Seoul, Korea

**Keywords:** Cognitive training, Memory, Spaced retrieval, Mild cognitive impairment, Randomized controlled trial, Computer, Tablet

## Abstract

**Background:**

Spaced retrieval training (SRT) is a nonpharmacological intervention for mild cognitive impairment (MCI) and dementia that trains the learning and retention of target information by recalling it over increasingly long intervals. We recently developed the Ubiquitous Spaced Retrieval-based Memory Advancement and Rehabilitation Training (USMART) program as a convenient, self-administered tablet-based SRT program. We also demonstrated the utility of USMART for improving memory in individuals with MCI through an open-label uncontrolled trial.

**Methods:**

This study had an open-label, single-blind, randomized, controlled, two-period crossover design. Fifty patients with MCI were randomized into USMART–usual care and usual care–USMART treatment sequences. USMART was completed or usual care was provided biweekly over a 4-week treatment period with a 2-week washout period between treatment periods. Primary outcome measures included the Word List Memory Test, Word List Recall Test (WLRT), and Word List Recognition Test. Outcomes were measured at baseline, week 5, and week 11 by raters who were blinded to intervention type. An intention-to-treat analysis and linear mixed modeling were used.

**Results:**

Of 50 randomized participants, 41 completed the study (18% dropout rate). The USMART group had larger improvements in WLRT score (effect size = 0.49, *p* = 0.031) than the usual care group. There were no significant differences in other primary or secondary measures between the USMART and usual care groups. Moreover, no USMART-related adverse events were reported.

**Conclusions:**

The 4-week USMART modestly improved information retrieval in older people with MCI, and was well accepted with minimal technical support.

**Trial registration:**

ClinicalTrials.gov NCT01688128. Registered 12 September 2012.

## Background

Mild cognitive impairment (MCI) represents an at-risk stage of cognitive decline between normal aging and dementia [1]. Given the absence of an approved pharmacological treatment for MCI [2], clinical research has advocated the use of several nonpharmacological interventions designed to optimize patient cognition, affect, and global functioning [3–6]. Spaced retrieval training (SRT) is one such intervention that trains the learning and retention of target information by recalling it over increasingly long intervals [7]. In previous research, SRT was found to improve prospective memory [8] and the capacity to learn face–name associations [9] in patients with MCI. SRT also improves semantic memory and behavioral strategies in patients with dementia [10, 11]. Studies have speculated that SRT improves learning through a combination of ecologically valid priming, spacing effect, conditioning, and errorless learning [11, 12]. In previous work by our group, we developed the 24-session Spaced Retrieval-based Memory Advancement and Rehabilitation Training (SMART) program and demonstrated its ability to improve memory retention spans in patients with very mild-to-mild Alzheimer’s disease (AD) through an open-label uncontrolled trial [13]. Expanded retention spans induced by the SMART program were maintained for different sets of target information, indicating that the effects of SMART on memory may be generalized [13, 14].

Information technologies are increasingly incorporated into therapeutic strategies to improve the accessibility and effectiveness of nonpharmacological interventions. Computer-based interventions enable the standardization and individualization of interventions, the unobtrusive real-time monitoring of cognitive performance, adjustment of the level of intervention, and reductions in personnel and implementation costs [15]. Computer-based cognitive training has been successfully delivered to old adults with normal cognition, MCI, and AD [15], and was effective in improving global cognition, selective cognitive domains, and psychosocial functioning of patients with MCI [16].

SRT was also delivered successfully to patients with dementia as a program on computers and tablets [17, 18]. A study of computer-assisted SRT for face–name associations in participants with mild to moderate dementia reported that 20 of 23 patients succeeded in learning novel and familiar names and biographical information over 32 minutes. Among these 20 participants, 17 patients showed transfer of familiar names from the training sessions to real-life interactions, and 19 patients retained the learned names until 6 weeks after the SRT [17]. In our previous work, we transformed the SMART program into a tablet-based application called the ubiquitous SMART (USMART) program. This program was self-administered by the patients with MCI or early dementia without the support of a trained therapist, and was effective in improving memory in patients with MCI in an open-label uncontrolled trial [14].

In the present study, we aimed to validate the efficacy of USMART on memory function in patients with MCI in a randomized controlled trial (RCT).

## Methods

### Subjects

We enrolled a total of 50 patients with MCI (10 amnestic single domain type, 25 amnestic multiple domains type, 12 nonamnestic single domain type, and 3 nonamnestic multiple domains type). Patients were recruited from the dementia clinic of Seoul National University Bundang Hospital (SNUBH) (*n* = 28) and the Korean Longitudinal Study on Cognitive Aging and Dementia (KLOSCAD) [19] (*n* = 22) between August 2014 and October 2014. The KLOSCAD is a population-based prospective older people cohort study on cognitive aging and dementia that was launched in 2009.

Research geropsychiatrists with expertise in dementia research evaluated each patient using the Korean version of the Consortium to Establish a Registry for Alzheimer’s Disease (CERAD-K) [20]. Research neuropsychologists administered the Korean version of the CERAD Neuropsychological Assessment Battery (CERAD-K-N) [20], the Frontal Assessment Battery (FAB) [21] and the Digit Span Test (DST) [22]. The CERAD-K-N [23] consists of nine neuropsychological tests, including the Categorical Fluency Test (CFT), the Modified Boston Naming Test (mBNT), the Mini Mental Status Examination (MMSE), the Word List Memory Test (WLMT), the Constructional Praxis Test (CPT), the Word List Recall Test (WLRT), the Word List Recognition Test (WLRcT), the Constructional Recall Test (CRT), and the Trail Making Test A (TMT-A).

MCI was diagnosed according to the revised diagnostic criteria for MCI proposed by the International Working Group on MCI [24]. Cognitive concerns were confirmed by the clinicians, who referred to self-and/or informant-reported cognitive decline. We ascertained the presence of objective cognitive impairment if a participant had a score less than −1.0 standard deviation (SD) of the age-, gender-, and education-adjusted norm for elderly Koreans on any of the 11 neuropsychological tests (FAB, DST forward, DST backward, CFT, mBNT, WLMT, CPT, WLRT, WLRcT, CRT, and TMT-A). Amnestic-type MCI was defined by impairment in any of the four memory tests (WLMT, WLRT, WLRcT, and CRT). We required that basic activities of daily living were preserved, and that impairment in complex instrumental functions were insufficient for a diagnosis of dementia. This level of functional impairment was determined by the clinical judgment of research geropsychiatrists and confirmed in case conferences by research geropsychiatrists. Patients with dementia were diagnosed according to the *Diagnostic and Statistical Manual of Mental Disorders*, Fourth Edition (DSM-IV) criteria [25] and excluded from the study. We also excluded patients with major psychiatric disorders listed in Axis I of the DSM-IV [25], any neurological disorders that could affect cognitive function, any physical condition that could preclude regular attendance and full intervention-program participation, and illiteracy. We did not exclude patients who were regularly taking stable doses of cognitive enhancers, sedatives, or antidepressants for at least 3 months before the study; these patients were instructed to maintain their medication regimens for the duration of the trial.

All participants were fully informed of the study protocol, and provided written informed consent, signed by the subjects or their legal guardians.

### Study design

**Fig. 1:**
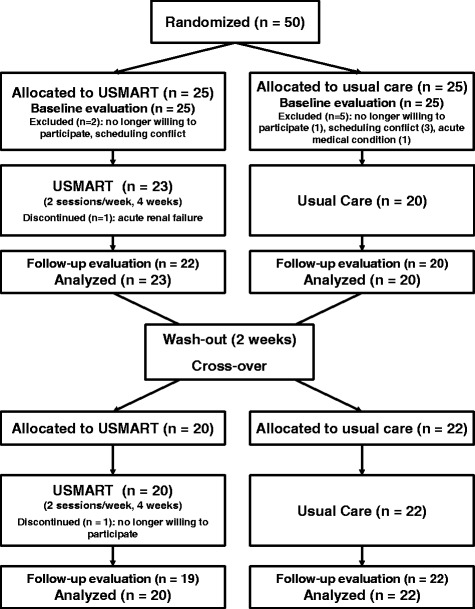
Trial flow chart. *n* number of subjects, *USMART* Ubiquitous Spaced Retrieval-based Memory Advancement and Rehabilitation Training

This study was an open-label, single-blind, randomized, controlled, two-period crossover trial (clinicaltrials.gov NCT01688128) investigating the efficacy of USMART in patients with MCI. Randomization was performed by the Medical Research Collaborating Center at SNUBH, who had no contact with patients or caregivers. A random code table and permuted-block randomization with varying block sizes [26] were used in SAS software, version 9.2 (SAS Institute Inc., Cary, NC, USA). The allocation sequence was produced independently and concealed until patients had entered the trial. The trial consisted of two 4-week periods that were crossed over. During the first period, patients were randomized to receive either USMART (*n* = 25) or usual care (*n* = 25). After a 2-week washout period, the groups were crossed over to receive the alternative treatment for 4 weeks. Clinical and neuropsychological characteristics were assessed at the beginning (week 0), the end of the first treatment period (week 5), and the end of the second treatment period (week 11) by raters who were blinded to intervention type (Fig. 1). This study protocol was approved by the Institutional Review Board of SNUBH (no. E-1207/162-001).

### Interventions

The USMART program was developed by transforming SMART, an offline face-to-face spaced retrieval-based memory training program, into a self-administered application on an iPad tablet [14]. Participants self-administered the USMART program for 30 min per session, twice per week, over the 4-week intervention period. In each session, the retrieval interval was sequentially doubled from 0.75 to 12 min (i.e., 0.75, 1.5, 3, 6, and 12 min). If the participant succeeded in recalling a given set of words within 12 min in two consecutive sessions, the number of words to be memorized within a session was automatically sequentially increased in the following session from one to five words. All procedures during USMART were guided by automatic verbal messages from the application [14]. In order to adhere to the study protocol, an occupational therapist was assigned to each participant, visited the participant at every training session with a tablet that had USMART installed, and took the tablet back after each training session. In each session, the assigned occupational therapist encouraged the participant to practice USMART, but did not provide any assistance for using the USMART application. We gathered the training records of the participants automatically via a web portal service.

### Outcome measures

The primary outcome measures were WLMT, WLRT, and WLRcT scores [20]. Secondary outcome measures included the Subjective Memory Complaint Questionnaire (SMCQ) which measures subjective memory complaints [27], the Geriatric Depression Scale (GDS) which measures the severity of depressive mood [28], and the MMSE which measures the level of global cognition [29]. All outcome measures were evaluated at weeks 0, 5, and 11 by trained research psychologists who were blinded to allocation information (Fig. 1).

### Sample size and statistical analyses

Assuming an attrition rate of 20%, a sample size of 50 patients (25 per treatment arm) would provide more than 80% power to detect a 2.5-point mean difference in WLRT with a SD of 2.53 (as in the Korean validation study [20]) at a two-sided type-I error of 0.05.

The intent-to-treat (ITT) population was defined as all randomized patients who provided at least one baseline efficacy assessment and attended at least one treatment session; this population was used for our primary efficacy evaluation. Five-week and 11-week last-observation-carried-forward analyses were performed for missing values. Baseline characteristics were summarized according to treatment sequence and compared using unpaired *t* tests for continuous variables and chi-squared tests for categorical variables. The effects of USMART on memory function, global cognition, and depressive mood were assessed using a linear mixed model for a repeated-measures covariance pattern model with compound symmetry within subjects. Period and treatment were included in the model as fixed effects, and patients were included in the model as a random effect. The models were adjusted for age, gender, education, and current cognitive-enhancer use. Effect sizes were calculated as described by Cohen [30]. For all analyses, two-sided *p* < 0.05 was considered statistically significant. All statistical analyses were performed using Predictive Analytics Software Statistics version 18.0.0 software (2009; SPSS, Inc., Chicago, IL, USA).

## Results

### Participants

Of 50 randomized participants, 43 completed the first intervention period (Fig. 1). Two patients from the USMART–usual care sequence and five patients from the usual care–USMART sequence did not enter the first intervention period because they were no longer willing to participate (*n* = 2), had an acute medical condition (*n* = 1), or had a scheduling conflict (*n* = 4). There were no statistically significant differences in the demographic or clinical characteristics of participants in the USMART–usual care sequence (*n* = 23) vs those in the usual care–USMART sequence (*n* = 20) in the ITT population (Table 1). After study initiation, one participant in the USMART–usual care sequence dropped out after session 5 of the first period because of acute renal failure. In the second period, one participant from the USMART–usual care sequence dropped because they were no longer willing to participate after session 4. There were no adverse events related to USMART during the first and second periods, and there were no dropouts due to difficulty operating the USMART application.

**Table 1:** Baseline demographic and clinical characteristics of participants

Characteristic	All participants	Sequence 1^a^	Sequence 2^b^	*p* value*
	(*n* = 43)	(*n* = 23)	(*n* = 20)	
Age (years)	74.01 ± 5.53	73.74 ± 4.84	74.50 ± 6.44	0.668
Female	20 (46.5%)	10 (43.5%)	10 (50.0%)	0.669
Educational level (years)	13.22 ± 3.33	13.52 ± 3.20	12.70 ± 3.69	0.443
Cognitive enhancer use	19 (44.2%)	10 (43.5%)	9 (45.0%)	0.128
WLMT	16.56 ± 4.41	15.83 ± 4.49	17.40 ± 4.28	0.247
WLRT	4.44 ± 2.37	4.30 ± 2.48	4.60 ± 2.30	0.687
WLRcT	8.47 ± 1.82	8.70 ± 1.49	8.20 ± 2.14	0.379
SMCQ	6.51 ± 3.15	5.87 ± 3.22	7.25 ± 2.97	0.152
GDS	9.28 ± 6.78	9.96 ± 7.46	8.50 ± 6.00	0.483
MMSE	25.12 ± 2.88	25.70 ± 3.17	24.45 ± 2.42	0.152

Data shown as mean ± standard deviation or *n* (%)

*Unpaired *t* tests for continuous variables and chi-squared tests for categorical variables

*GDS* Geriatric Depression Scale, *MMSE* Mini Mental State Examination, *n* number of subjects, *SMCQ* Subjective Memory Complaint Questionnaire, *USMART* Ubiquitous Spaced Retrieval-based Memory Advancement and Rehabilitation Training, *WLMT* Word List Memory Test, *WLRcT* Word List Recognition Test, *WLRT* Word List Recall Test

^a^Participants in the USMART–usual care sequence

^b^Participants in the usual care–USMART sequence

### Efficacy

Changes in WLMT, WLRT, WLRcT, SMCQ, GDS, and MMSE scores are presented in Table 2. USMART was more beneficial than usual care for improving measures of memory recall function (1.12 ± 1.56 vs 0.36 ± 1.56 points, respectively; effect size = 0.49; *p* = 0.031) for the WMRT. In the USMART period, 62.8% of participants showed improvement in WLRT score, whereas in the usual care period, only 35.7% of participants showed improvement (*p* = 0.013). Among secondary measures, there were no significant differences in changes in GDS, SMCQ, or MMSE scores between the USMART and usual care periods. Carryover effects of the first period were not significant for any primary or secondary outcome measures (*p* > 0.1).

**Table 2:** Efficacy of USMART vs usual care

	USMART (*n* = 43)			Usual care (*n* = 42)			Statistics^a^	
Outcome measure	Prior	after	Change	Prior	After	Change	*p* value	Cohen's *d* (95% CI)
WLMT	16.91 ± 4.49	18.81 ± 5.12	1.91 ± 2.68	17.79 ± 4.48	19.12 ± 4.49	1.33 ± 3.21	0.351	0.20 (−0.60–1.17)
WLRT	4.63 ± 2.31	5.74 ± 2.26	1.12 ± 1.56	5.14 ± 2.35	5.50 ± 2.19	0.36 ± 1.56	0.031	0.49 (0.03–0.96)
WLRcT	8.56 ± 1.82	8.95 ± 1.38	0.40 ± 1.40	8.62 ± 1.83	8.52 ± 1.93	−0.10 ± 1.45	0.229	0.36 (−0.06–0.79)
SMCQ	6.07 ± 3.13	5.81 ± 3.34	−0.26 ± 2.04	5.90 ± 3.32	5.62 ± 3.34	−0.29 ± 2.09	0.705	0.01 (−0.60–0.65)
GDS	9.23 ± 6.80	8.49 ± 6.85	−0.74 ± 4.25	8.60 ± 6.80	8.17 ± 6.8	−0.43 ± 4.04	0.799	−0.08 (−1.35–1.15)
MMSE	25.49 ± 3.40	26.37 ± 2.99	0.88 ± 2.89	25.83 ± 2.92	25.76 ± 3.28	−0.07 ± 2.31	0.118	0.37 (−0.50–1.07)

Data shown as mean ± standard deviation

*CI* confidence interval, *GDS* Geriatric Depression Scale, *MMSE* Mini Mental State Examination, *n* number of subjects, *SMCQ* Subjective Memory Complaint Questionnaire, *USMART* Ubiquitous Spaced Retrieval-based Memory Advancement and Rehabilitation Training, *WLMT* Word List Memory Test, *WLRcT* Word List Recognition Test, *WLRT* Word List Recall Test

^a^By linear mixed model adjusted for age, gender, education, and current cognitive-enhancer use

## Discussion

In this study, we found that biweekly self-administration of USMART over a 4-week period was more effective than usual care for improving memory function in patients with MCI. Operation of the USMART application seemed to be simple and easy for patients with MCI, because no patients dropped out due to difficulty with the application. To our knowledge, this is the first RCT on the efficacy of self-administered computerized SRT.

Previous RCTs indicate that SRT is effective in improving training content-associated tests in various cognitive domains and functions such as personal information recall [31], name–face association [31], instrumental activities of daily living [32], and problematic behaviors such as eating difficulty [33]. However, these studies did not find a generalization of the SRT effects to neuropsychological tests independent of training content in the SRT [31, 32], except in a measure of attention [31].

In the current RCT, USMART improved performance in patients with MCI on the WLRT, which uses a set of words that are different from those in the training content of USMART. According to Valenzuela and Sachdev [34], generalization can happen at multiple levels. These include transfer to nontrained tasks in the same cognitive domain, transfer to nontrained tasks in other cognitive domains, transfer to global measures of general cognitive ability, and transfer to measures of general function. The transfer to nontrained tasks in the same cognitive domain is the lowest level and that to measures of general function is the highest level in the hierarchy of generalization [34]. Therefore, the current study directly shows that the efficacy of USMART can be transferred to other nontrained tasks in the same cognitive domain (i.e., WLRT), which is the lowest level of generalization. Furthermore, USMART could not improve MMSE (a measure of global cognition) and SMCQ (a measure of function) scores. This indicates that the efficacy of USMART cannot be transferred to general cognitive ability or function. Although we did not measure performance in other cognitive domains in the current study, the efficacy of USMART may not be transferred to nontrained measures of other cognitive domains. This is because these effects were not transferred to the WLMT and WLRcT, which are measures of memory function, although they test different processes of memory than the WLRT. However, the generalizability of USMART should be investigated in future research, because the duration of treatment in this study was short and the sample size was small. In addition, whether improvements due to SRT using different types of memory, such as stories or designs, can also be transferred to other nontrained tasks in the same cognitive domain warrants further research.

The effect size of USMART on the WLRT was modest [30]. One recent meta-analysis reported that the effect of computerized cognitive training on cognition in individuals with MCI is moderate [16]. Another systematic review reported that the effect sizes of memory strategy trainings ranged from –1.18 to 0.88 in individuals with MCI [35]. Although there have been no clinical trials to investigate the effect size of SRT in patients with MCI, a meta-analysis of the effects of SRT on semantic memory in patients with mild AD reported that the effect sizes varied substantially from 0.67 to 37.97 across studies [10]. The authors attributed the wide variation in the effect size of SRT between studies to differences in protocols for SRT, sizes of study samples, outcome measures, and quality of the clinical trials [10].

Several studies failed to show that the effects of SRT generalize to standardized neuropsychological tests of memory function in the patients with AD or dementia [13, 31]. Conventional neuropsychological tests for memory function require more cognitive effort and explicit memory than the new learning in SRT, which mainly uses implicit memory [36]. Patients with AD have more severe explicit memory impairments than those with MCI. Therefore, the efficacy of SRT is less likely to be transferred to neuropsychological tests in the same cognitive domain in patients with AD when compared to those with MCI.

Before USMART, SRTs were implemented in computers [17] or mobile devices [37]. Computerized cognitive training received significant attention as a more cost-effective and accessible option compared to traditional paper-and-pencil cognitive training [38]. Tablet computers in particular have more advantages in their portability and flexibility for delivering a variety of tasks [39]. Limited adaptability to new technology is a common concern for the application of computerized cognitive training in older patients and patients with cognitive impairment. However, two studies have already shown that cognitive training using iPads is effective in improving episodic memory and processing speed in older people [39, 40]. The older individuals with MCI successfully used USMART with minimal technical support in the current study, and those with early-stage AD, all of whom were first-time mobile device users, also successfully used the tablet-based SRT in a previous study [18]. Therefore, a well-designed mobile user interface may become quite applicable as a platform for delivering cognitive training, such as SRT, to older individuals or patients with cognitive impairment.

**Fig. 2:**
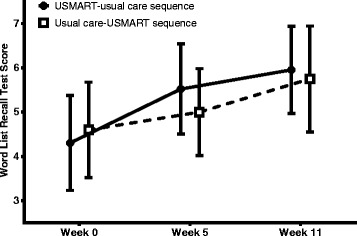
WLRT scores during the trial according to the treatment sequence. *Bar*: 95% confidence interval. *Week 0* the beginning (baseline), *Week 5* the end of the first treatment period, *Week 11* the end of the second treatment period, *USMART* Ubiquitous Spaced Retrieval-based Memory Advancement and Rehabilitation Training

The present study had several limitations. First, this study was not double-blinded. Second, we employed usual care as a control instead of mock therapy. Third, our sample size was relatively small, but was notably consistent with most previous RCTs on SRT [9, 31, 32, 41]. Fourth, subtypes and causes of MCI were not adjusted for in the analysis. Fifth, the duration of treatment was too short to test the efficacy of USMART on general cognition or function. The efficacy of USMART increased with increasing numbers of training sessions in our previous uncontrolled trial [14]. In addition, the differences in outcome measures between the USMART group and the usual care group might have been attenuated due to learning effects, because the intervals between the outcome measurements were relatively short (Fig. 2). Sixth, we did not evaluate how long the improvement in the WLRT lasted.

## Conclusions

The 4-week USMART modestly improved information retrieval in older people with MCI, and was well accepted with minimal technical support.

## Abbreviations

AD: Alzheimer’s disease
CERAD-K: Consortium to Establish a Registry for Alzheimer’s Disease
CERAD-K-N: Korean version of the CERAD Neuropsychological Assessment Battery
CFT: Categorical Fluency test
CPT: Constructional Praxis Test
CRT: Constructional Recall Test
DSM-IV: *Diagnostic and Statistical Manual of Mental Disorders*, Fourth Edition
DST: Digit Span Test
FAB: Frontal Assessment Battery
GDS: Geriatric Depression Scale
ITT: Intent-to-treat
KLOSCAD: Korean Longitudinal Study on Cognitive Aging and Dementia
mBNT: Modified Boston Naming Test
MCI: Mild cognitive impairment
MMSE: Mini Mental Status Examination
RCT: Randomized controlled trial
SD: Standard deviation
SMART: Spaced Retrieval-based Memory Advancement and Rehabilitation Training
SMCQ: Subjective Memory Complaint Questionnaire
SNUBH: Seoul National University Bundang Hospital
SRT: Spaced retrieval training
TMT-A: Trail Making Test A
USMART: Ubiquitous Spaced Retrieval-based Memory Advancement and Rehabilitation Training
WLMT: Word List Memory Test
WLRcT: Word List Recognition Test
WLRT: Word List Recall Test
